# Circulating interleukin-6 and cancer: A meta-analysis using Mendelian randomization

**DOI:** 10.1038/srep11394

**Published:** 2015-06-22

**Authors:** Geng Tian, Jia Mi, Xiaodan Wei, Dongmei Zhao, Lingyan Qiao, Chunhua Yang, Xianglin Li, Shuping Zhang, Xuri Li, Bin Wang

**Affiliations:** 1 Medicine and Pharmacy Research Center, Binzhou Medical University, Laishan District, Yantai, Shandong, China; 2 Institute of Anatomy, Binzhou Medical University, Laishan District, Yantai, Shandong, China; 3 Clinic Institute, Binzhou Medical University, Laishan District, Yantai, Shandong, China; 4 Institute of Pharmacology, Binzhou Medical University, Laishan District, Yantai, Shandong, China; 5 Institute of Molecular Imaging, Binzhou Medical University, Laishan District, Yantai, Shandong, China

## Abstract

Interleukin-6 (IL-6) plays a contributory role in the progression and severity of many forms of cancer; it however remains unclear whether the relevance between circulating IL-6 and cancer is causal. We therefore meta-analyzed published articles in this regard using *IL-6* gene -174G/C variant as an instrument. Seventy-eight and six articles were eligible for the association of -174G/C variant with cancer and circulating IL-6, respectively. Overall analyses failed to identify any significance between -174G/C and cancer risk. In Asians, carriers of the -174CC genotype had an 1.95-fold increased cancer risk compared with the -174GG genotype carriers (P = 0.009). By cancer type, significance was only attained for liver cancer with the -174C allele conferring a reduced risk under allelic (odds ratio or OR = 0.74; P = 0.001), homozygous genotypic (OR = 0.59; P = 0.029) and dominant (OR = 0.67; P = 0.004) models. Carriers of the -174CC genotype (weighted mean difference or WMD = −4.23 pg/mL; P < 0.001) and -174C allele (WMD = −3.43 pg/mL; P < 0.001) had circulating IL-6 reduced significantly compared with the non-carriers. In further Mendelian randomization analysis, a reduction of 1 pg/mL in circulating IL-6 was significantly associated with an 12% reduced risk of liver cancer. Long-term genetically-reduced circulating IL-6 might be causally associated with a lower risk of liver cancer.

As a multifactorial cytokine, interleukin-6 (IL-6) is widely believed to play a role in the progression and severity of many forms of cancer. Several observational studies have suggested that circulating IL-6 can explain inter-individual variability in predisposition to cancer. Heikkila and colleagues have written an excellent review, highlighting the involvement of elevated circulating IL-6 in human carcinogenesis^1^. However, it remains unclear whether the relevance between circulating IL-6 and cancer is causal as the issue of confounding or reverse causation is often unavoidable in observational epidemiology.

Ideally, randomized controlled trial of the intervention that alters circulating IL-6 is considered as the gold standard for evaluating this causal relevance, but in some circumstances it is neither practical nor ethical to randomize human beings for such trials. As an alternative, a more promising method termed as ‘Mendelian randomization’ has been developed to exploit the impact of long-term exposure differences on disease risk by using a genetic instrument to account for the potential biases due to confounding and reverse causation^2^. Like a randomized controlled trial, Mendelian randomization randomizes genotypes at conception according to Mendel’s second law^3^. This method has been successfully applied to a variety of genetic exposures such as obesity and alcohol consumption in causal susceptibility to cancer^4^.

The most immediate determinant of circulating IL-6 is its encoding gene *IL-6*. The genomic sequence of *IL-6* gene is highly polymorphic and one of the most frequently evaluated variants is -174G/C (rs1800795) in the promoter region^5^,^6^. *In vitro* studies have found the allele-specific impact of -174G/C variant on *IL-6* gene promoter activity, with the -174C allele corresponding to lower expression level^7^. Besides, carriers of the -174G allele had a higher level of circulating IL-6 than those with the -174CC genotype^8^,^9^. Based on these observations, we thereby develop a completing hypothesis that the association between circulating IL-6 and cancer is causally related. To test this hypothesis, we employed Mendelian randomization technique to meta-analyze all available published articles in this regard by using *IL-6* gene -174G/C variant as an instrument.

## Results

### Eligible articles

**Figure 1:**
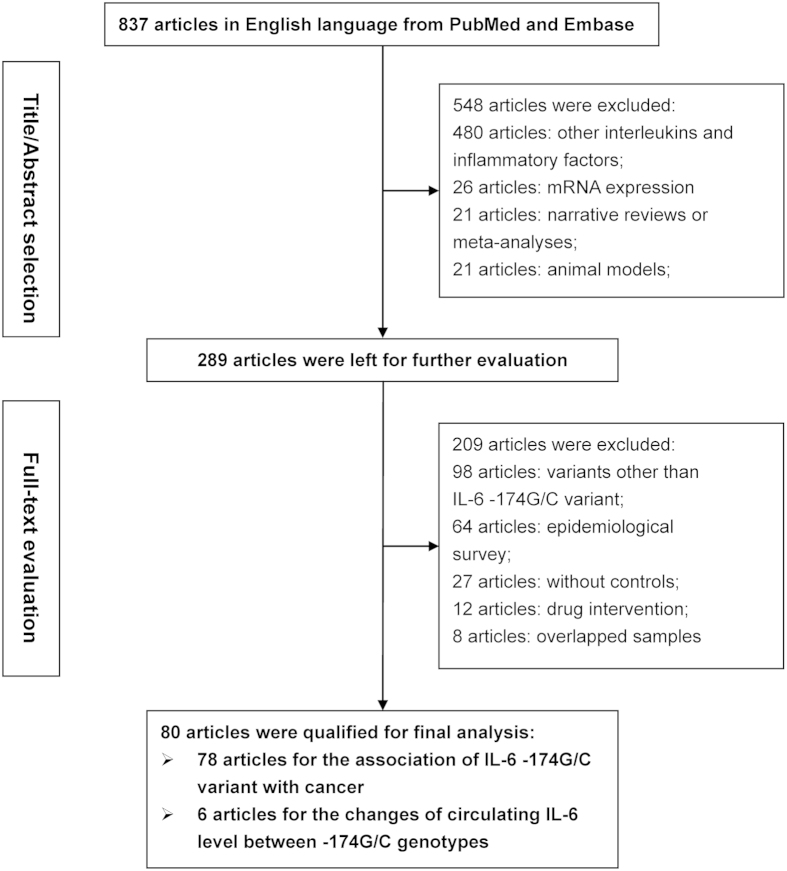
Flow diagram of search strategy and study selection.

The selection process of articles is schematized in Fig. 1. A total of 837 potentially relevant articles were identified after an initial literature search and 80 of them written in English language were finally analyzed^5^,^6^,^8^,^9^,^10^,^11^,^12^,^13^,^14^,^15^,^16^,^17^,^18^,^19^,^20^,^21^,^22^,^23^,^24^,^25^,^26^,^27^,^28^,^29^,^30^,^31^,^32^,^33^,^34^,^35^,^36^,^37^,^38^,^39^,^40^,^41^,^42^,^43^,^44^,^45^,^46^,^47^,^48^,^49^,^50^,^51^,^52^,^53^,^54^,^55^,^56^,^57^,^58^,^59^,^60^,^61^,^62^,^63^,^64^,^65^,^66^,^67^,^68^,^69^,^70^,^71^,^72^,^73^,^74^,^75^,^76^,^77^,^78^,^79^,^80^,^81^,^82^,^83^,^84^. Article involving more than one independent study group was analyzed separately. Altogether, 78 articles with 87 study groups (45569 cancer patients and 57990 controls) were eligible for the association between *IL-6* gene -174G/C variant and cancer^6^,^9^,^10^,^11^,^12^,^13^,^14^,^15^,^16^,^17^,^18^,^19^,^20^,^21^,^22^,^23^,^24^,^25^,^26^,^27^,^28^,^29^,^30^,^31^,^32^,^33^,^34^,^35^,^36^,^37^,^38^,^39^,^40^,^41^,^42^,^43^,^44^,^45^,^46^,^47^,^48^,^49^,^50^,^51^,^52^,^53^,^54^,^55^,^56^,^57^,^58^,^59^,^60^,^61^,^62^,^63^,^64^,^65^,^66^,^67^,^68^,^69^,^70^,^71^,^72^,^73^,^74^,^75^,^76^,^77^,^78^,^79^,^80^,^81^,^82^,^83^,^84^, and 6 articles with 9 study groups (1727 study subjects) were eligible for circulating IL-6 changes across -174G/C genotypes^5^,^8^,^9^,^13^,^16^,^52^.

### Study characteristics

Table 1 summarizes the baseline characteristics of 87 eligible study groups for the association between *IL-6* gene -174G/C variant and cancer. Supplementary Table S1 provides the quality assessment of 87 study groups and the genotype distributions of -174G/C variant. The quality score ranged from 4 to 11 and was averaged at 8.37. Cancer patients were older than controls (mean age: 59.26 versus 55.01 years, P = 0.0007). The percentage of smokers was slightly higher in cancer patients than healthy controls (42.24% versus 33.06%, P = 0.038). No significance was observed in mean body mass index and the percentages of male gender and drinkers between the two groups (P > 0.05).

Forty-six of 87 study groups were conducted in Caucasians, 31 in mixed populations, 9 in Asians and 1 in African-Americans. Sixteen study groups focused on colorectal cancer, 14 groups on breast cancer, 8 groups on gastric cancer, 7 groups on prostate cancer, 6 groups on myeloma, 5 groups respectively on lung and liver cancers, 4 groups on lymphoma, 3 groups on oral cancer, 2 groups respectively on esophageal, basal cell, bladder, cervical, leukatmia, gallbladder and thyroid cancers, 1 group respectively on melanoma, ovarian, renal cell, leiomyoma and pancreatic cancers. Thirty-three of 87 study groups had total sample size of at least 500. Age was reported to be matched in 60 study groups and unmatched in 4 study groups between cancer patients and controls. Fifty-seven study groups had controls enrolled from general populations and 30 study groups from hospitals. Fifty-nine and 28 study groups followed a retrospective and prospective design, respectively. Sixty-five of 87 study groups had genotype distributions of -174G/C variant in Hardy–Weinberg equilibrium at a significance level of 5%, 19 study groups in Hardy–Weinberg disequilibrium, and 3 study groups without mutation. The frequency of *IL-6* gene -174C allele ranged from 0.0% to 52.94% in cancer patients and from 0.0% to 51.15% in healthy controls. By contrast, this frequency was significantly lower in Asians than in Caucasians (cancer patients: 0.0% to 28.0% versus 17.90% to 52.94%; healthy controls: 0.0% to 26.75% versus 13.33% to 51.15%).

Table 2 summarizes mean circulating IL-6 across -174G/C genotypes. All study groups involved Caucasians except for one with mixed descents. Seven of 9 study groups provided circulating IL-6 in cancer patients, and 1 study group respectively in healthy controls and in combined patients and controls.

### Prediction of -174G/C variant for cancer risk

**Figure 2:**
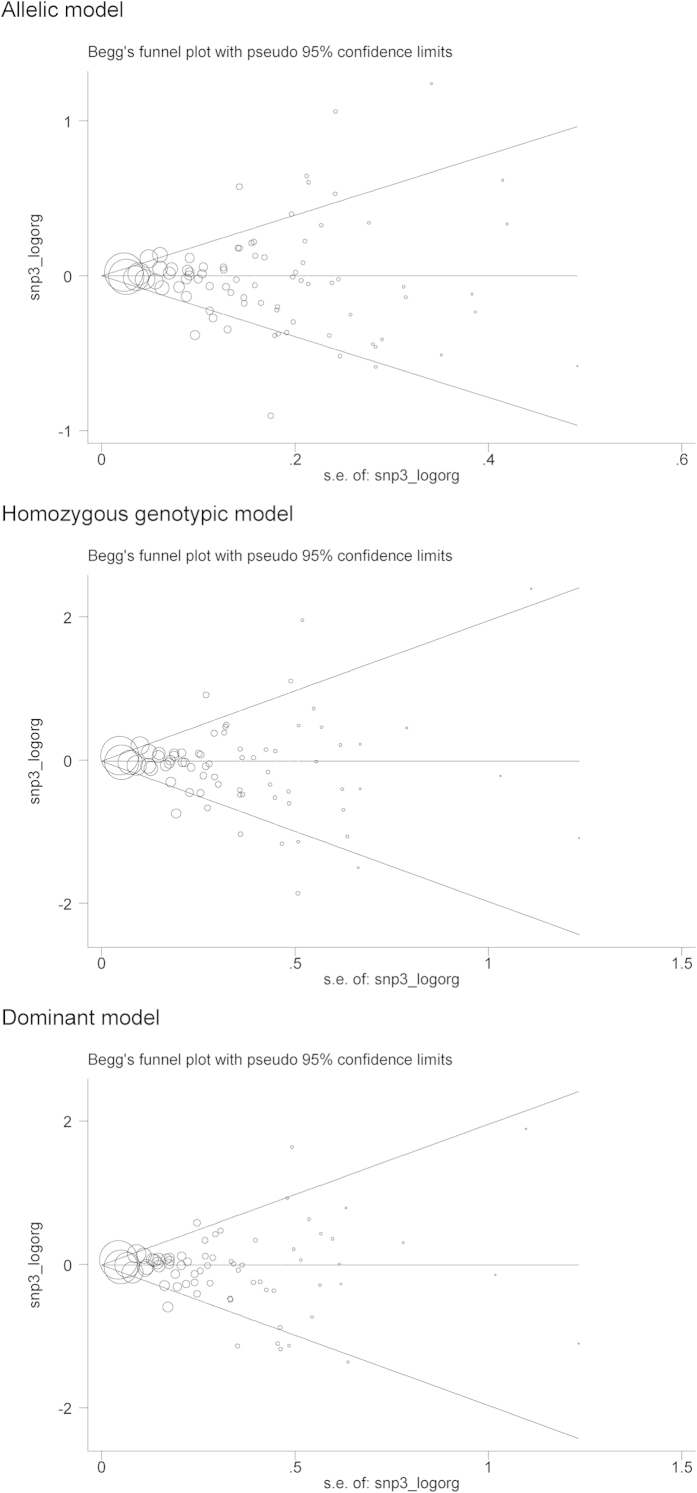
The Begg’s funnel plots of *IL-6* gene -174G/C variant for cancer under three genetic models.

Overall and subgroup estimates of *IL-6* gene -174G/C variant for cancer risk are provided in Table 3. Overall analyses failed to identify any significance for the -174C allele under allelic (OR = 1.02; 95% CI: 0.98 to 1.07; P = 0.290), homozygous genotypic (OR = 1.07; 95% CI: 0.99 to 1.16; P = 0.103) and dominant (OR = 1.02; 95% CI: 0.97 to 1.08; P = 0.465) models, with moderate heterogeneity (*I*^2^ = 66.6%, 54.0% and 65.1%, respectively). There was no indication of publication bias for three genetic models except for the homozygous genotypic model (Egger’s test: P = 0.075) (Fig. 2). After restricting study groups with Hardy–Weinberg equilibrium, there was no material change in effect estimates.

Potential sources of heterogeneity were exploited by subgroup analyses according to ethnicity, cancer type, matching condition, source of controls, study design and sample size, respectively (Table 3). To avoid chance results, only subgroups with three or more study groups were considered in this meta-analysis. By ethnicity, no significance was attained in Caucasians under three genetic models, and contrastingly Asian carriers of -174CC genotype were observed to have 1.95-fold increased cancer risk compared with those with the -174GG genotype (95% CI: 1.95; 1.19 to 3.20; P = 0.009), even after the Bonferroni correction to control for the multiple testing (P < 0.05/3, here 3 refers to the total number of subgroups by ethnicity). There was no heterogeneity (*I*^2^ = 17.9%) for this significant association.

By cancer type, effect estimates were significant only for liver cancer with the -174C allele conferring a reduced risk under allelic (OR = 0.74; 95% CI: 0.61 to 0.89; P = 0.001), homozygous genotypic (OR = 0.59; 95% CI: 0.36 to 0.95; P = 0.029) and dominant (OR = 0.67; 95% CI: 0.52 to 0.88; P = 0.004) models, and this significance was less likely to be interpreted by heterogeneity (*I*^2^ = 0.0%, 0.0% and 21.5%, respectively). Even after the Bonferroni correction, significance was still preserved for the allelic and dominant models (P < 0.05/9, here 9 refers to the total number of subgroups by cancer type). Effect estimates of -174G/C variant for cancer by source of controls, study design and sample size did not deviate significantly from the unity under three genetic models (all P > 0.05), and there was no material improvement in heterogeneity within these subgroups.

### Changes of circulating IL-6 across -174G/C genotypes

**Figure 3:**
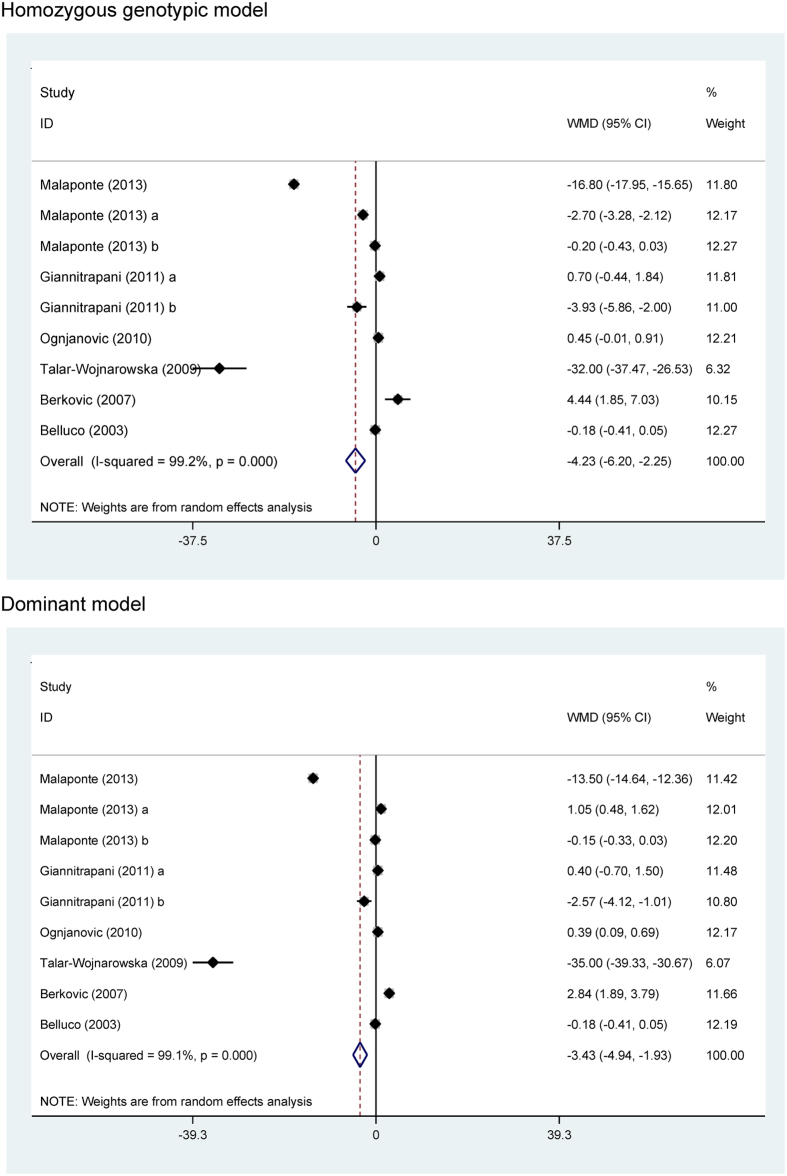
The funnel plots of circulating IL-6 changes across *IL-6* gene -174G/C genotypes under homozygous genotypic and dominant models.

Carriers of the -174CC genotype (WMD = −4.23 pg/mL; 95% CI: −6.20 to −2.25; P < 0.001) and -174C allele (-174CC and -174GC genotypes) (WMD = −3.43 pg/mL; 95% CI: −4.94 to −1.93; P < 0.001) had significantly lowered circulating IL-6 when compared with the -174GG genotype carriers, yet with strong evidence of heterogeneity (*I*^2^ = 99.2% and 99.1%, respectively) (Fig. 3).

### Predicted causality of circulating IL-6 for cancer

Under the principles of Mendelian randomization, a reduction of 1 pg/mL in circulating IL-6 was significantly associated with an 12% reduced risk of liver cancer (95% CI: 0.64 to 0.99). However in Asians, this association was totally reversed with 1 pg/mL reduced circulating IL-6 corresponding to an 17% increased cancer risk (95% CI: 1.03 to 1.68). Considering that the unity was not included by above 95% CIs, it is safe to the reject the null hypothesis of none causal relevance between circulating IL-6 and certain cancer subtypes.

### Sensitivity analysis

Sensitivity analysis confirmed the overall differences in risk estimates for the prediction of *IL-6* gene -174G/C variant for cancer risk and circulating IL-6 changes between -174G/C genotypes in both direction and magnitude by sequentially omitting each study once at a time and computing differential estimates for the remaining studies.

## Discussion

In this meta-analysis of 80 qualified articles, we employed Mendelian randomization to test the completing hypothesis that the association between circulating IL-6 and cancer is causal. The most noteworthy finding of this study was that long-term genetically-reduced circulating IL-6 might be causally associated with a lower risk of liver cancer. To the authors’ knowledge, this is the first comprehensive meta-analysis assessing the impact of long-term differences in circulating IL-6 on cancer risk.

Currently, targeted anti-IL-6 antibody therapy has been successfully applied in several clinical trials and found to be well tolerated in cancer patients^85^. Evidence from epidemiological studies is accruing in favor of a contributory role of elevated circulating IL-6 in patients with advanced tumor stages of various cancers, such as non-small cell lung cancer, colorectal cancer and renal cell carcinoma^86^,^87^,^88^. Currently, whether the progression and severity of caner is due to elevated circulating IL-6 still remains an open question. Genetic association studies are deemed as more similar to randomized clinical trials than other types of observational epidemiological studies due to Mendelian randomization (Mendel’s second law)^89^. We therefore utilized Mendelian randomization to assess whether the relevance between circulating IL-6 and cancer is causal by selecting the most frequently evaluated variant -174G/C in *IL-6* gene as a genetic instrument to minimize residual confounding and reverse causation.

In this meta-analysis, risk estimates of *IL-6* gene -174G/C variant with cancer were heterozygous between Caucasians and Asians. Considering the multifactorial nature of cancer, divergent genetic backgrounds or linkage disequilibrium patterns might be the most likely explanation for such divergence^90^. This is well exemplified in the present study with regard to the frequency of -174C allele, which was exceedingly lower in Asians than in Caucasians (Table 1). Even in some Asian populations, the mutation rate of this allele was zero^40^,^67^,^81^. Generally, it is not uncommon for the same variant playing a different role in cancer susceptibility across different populations. This is the principal limitation of this Mendelian randomization meta-analysis, that is, if the other flanking variants within or near *IL-6* gene related to cancer risk are in linkage disequilibrium with -174G/C variant we have examined, this will confound our findings. What’s more, such confounding is difficult to exclude completely; however it is unlikely that it would explain our finding that *IL-6* gene -174CC genotype was associated with lowered circulating IL-6 without predicting a low cancer risk. There is also evidence that a variant may be in close linkage with another nearby causal locus in one ethnic population but not in another^91^. In view of this limitation, it is necessary to establish an ethnicity-specific database of candidate genes and variants in susceptibility to cancer^92^. Another limitation of this study is that excluding the pleiotropy of *IL-6* gene -174G/C variant seems impractical for us since data on other inflammatory factors across -174G/C genotypes are rarely provided from most eligible articles, necessitating further confirmation using additional genetic variants and/or exposure outcomes.

It is also worth noting that *IL-6* gene -174G/C variant exhibited heterozygous association with different forms of cancer in this meta-analysis. For example, the -174C allele was observed to confer a significantly protective effect against liver cancer, yet a risk effect for oral cancer with no attainable significance. The identification of -174G/C variant affecting the significant risk of liver cancer allowed us to employ Mendelian randomization to account for potential biases due to residual confounding and reverse causation. Consistent with the findings of other studies^8^,^9^, carriers of the -174C allele or -174CC genotype had lower circulating IL-6 than the non-carriers, supporting the plausibility of causal relevance between circulating IL-6 and liver cancer. Given the insufficient statistical power of this meta-analysis in some subgroups, far larger sample sizes than studied here will be required to produce enough power to evaluate the causality between circulating IL-6 and various forms of cancer.

Several limitations should be acknowledged in this meta-analysis. Firstly, only published articles were retrieved and the ‘grey’ literature (articles written in languages other than English) was not covered, leading to the possible existence of publication bias. However, the influence of publication bias on the gene-disease association is expected to result in an overestimation, rather than an underestimation. Secondly, as the majority of involved studies in this meta-analysis recruited cancer patients aged over 50 years for whom environmental factors are likely to contribute more prominently than a genetic component to the development of cancer, more large studies in a younger cancer population will of great interest. Thirdly, given the possible impact of drug regimens on circulating IL-6, the relationship between circulating IL-6 changes and IL-6 gene -174G/C genotypes might be biased, calling for further validation of this relationship in healthy controls. Fourthly, nearly all involved studies had circulating IL-6 measured only once, which cannot reflect its long-term level in the development of cancer. Fifthly, this meta-analysis was based on summarized data, rather than individual participant data, precluding further gene-to-environment interaction. Sixthly, only one variant in *IL-6* gene was selected, and investigation on other candidate genes or polymorphisms involved in IL-6 regulation was highly encouraged, leaving a challengeable task to test whether this variant integrated with other risk determinants will enhance cancer risk prediction. The jury therefore must refrain from drawing a firm conclusion until large, well-designed studies to confirm our findings.

To sum up, there is possible evidence for causal association between long-term genetically-reduced circulating IL-6 and reduced risk of liver cancer. For practical reasons, we hope that this study will advance our understanding of the role of circulating IL-6 leading to the progression and severity of liver cancer. Further studies to elucidate the specific role of IL-6 in cancer pathogenesis are required.

## Methods

The implementation of this meta-analysis complied with the guidelines outlined in the Preferred Reporting Items for Systematic Reviews and Meta-analyses (PRISMA) statement (Please see the supplementary PRISMA Checklist)^93^.

### Search strategy

PubMed and Embase (excerpta medica database) were searched for potentially relevant articles from the earliest possible year to August 4, 2014. The key terms included ‘interleukin-6’, ‘interleukin 6’, ‘IL6’, ‘IL-6’, ‘IL 6’, ‘cancer’, ‘carcinoma’, ‘neoplasia’, ‘tumor’, ‘adenoma’, ‘neoplasm’, ‘myeloma’, ‘melanoma’, ‘lymphoma’, ‘leukaemia’, ‘leiomyoma’, in combination with ‘level’, ‘concentration’, ‘polymorphism’, ‘variant’, ‘variation’, ‘mutation’, ‘SNP’. Citations from retrieved potential articles and reviews were also checked for eligibility.

The titles and abstracts of all retrieved relevant articles were independently reviewed by two investigators (Chunhua Yang and Xuri Li). In case of uncertainty for rejection, the full text and supplementary materials if available were downloaded to check whether information on the topic of interest was provided. If more than one article from a study group was published, data from the most recent or complete article were abstracted. The eligibility of each retrieved article was assessed in duplicate and independently by two authors (Chunhua Yang and Xuri Li). Any uncertainty over the eligibility was adjudicated by further joint inspection of the articles.

### Inclusion/exclusion criteria

Inclusion criteria were to test the hypothesis that *IL-6* gene -174G/C variant was associated with cancer or circulating IL-6 and to provide detailed genotype or allele counts of this variant between cancer patients and healthy controls or the mean levels of circulating IL-6 across -174G/C genotypes. Articles were excluded if they assessed the progression, severity, phenotypic modification and response to treatment or survival of cancer, or if they lacked patients or controls, or if they were conference abstracts or proceedings, case reports or series, editorials, narrative reviews, and non-English articles.

### Data extraction

Two authors (Chunhua Yang and Xuri Li) independently abstracted the following data from each qualified article according to a fixed protocol, including the first author’s last name, publication year, ethnicity, cancer type, matching condition, study design, sample size, the genotype or allele counts of the -174G/C variant between patients and controls, mean level of circulating IL-6 for each genotype carriers expressed as mean ± standard deviation, as well as some baseline characteristics of study populations where available, including age, gender, body mass index, the percentages of smokers and drinkers. The unit of circulating IL-6 was uniformly transferred into pg/mL in this meta-analysis.

### Quality assessment

Criteria for quality assessment of the association between *IL-6* gene -174G/C variant and cancer risk were in agreement with the standards formulated by Thakkinstian *et al.*^94^ There were 7 criteria in total and summarized as a quality score, ranging from 0 (the worst) to 12 (the best). Quality assessment was independently conducted by two authors (Chunhua Yang and Xuri Li), and any disagreement was resolved by consensus.

### Statistics

Risk estimates for the association of *IL-6* gene -174G/C variant with cancer were expressed as odds ratio (OR) and its corresponding 95% confidence interval (95% CI), and for the changes of circulating IL-6 between genotypes of this variant as weighted mean difference (WMD) and its 95% CI. Hardy–Weinberg equilibrium was tested by Chi-squared test. Differences at P < 0.05 were accepted as statistically significant. A random-effects model was employed to bring individual effect-size estimates together by using the DerSimonian and Laird method.

Heterogeneity between studies was quantified by the inconsistency index (*I*^2^) statistic, which ranges from 0% to 100%. This statistic is defined as the percentage of the observed between-study variability that is due to heterogeneity rather than chance. A threshold of over 50% for *I*^2^ statistic was treated as statistically significant heterogeneity.

Predefined subgroup analyses were explored to identify the potential sources of between-study heterogeneity according to ethnicity, cancer type, matching condition, source of controls (population-based controls and hospital-based controls), study design (prospective study and retrospective study) and sample size (<500 subjects and ≥500 subjects). To assess the contribution of each individual studies to pooled effect estimates, sensitivity analyses were undertaken by sequentially omitting each study one at a time and computing differential estimates for remaining studies.

Publication bias was assessed by the Begg’s funnel plot and the Egger regression asymmetry test. The Egger test can identify the asymmetry of funnel plots by determining whether the intercept deviates significantly from zero in regressing the standardized effect estimates against their precision. A value of P < 0.10 was used to indicate statistical significance for Egger’s test.

Risk estimates in Mendelian randomization analysis were calculated as the ratio of the coefficient of the association between *IL-6* gene -174G/C variant and cancer to that of the association between -174G/C variant genotypes and circulating IL-6 as a reflection of the potential causal impact of circulating IL-6 on cancer.

All statistical analyses described above were completed with the StataCorp STATA version 12.0.

## Additional Information

**How to cite this article**: Tian, G. *et al.* Circulating interleukin-6 and cancer: A meta-analysis using Mendelian randomization. *Sci. Rep.* **5**, 11394; doi: 10.1038/srep11394 (2015).

## Supplementary Material

Supplementary Information

## Figures and Tables

**Table 1:** The baseline characteristics of all study groups in this meta-analysis.

**Author (year)**	**Ethnicity**	**Cancer type**	**Match**	**Source of controls**	**Study design**	**Sample size**		**Age (years)**		**Males**		**BMI (kg/m**^**2**^)		**Smoking**		**Drinking**	
						**Cases**	**Cont’s**	**Cases**	**Cont’s**	**Cases**	**Cont’s**	**Cases**	**Cont’s**	**Cases**	**Cont’s**	**Cases**	**Cont’s**
Slattery (2014)	Mixed	Breast	NA	Population	Prosp.	3567	4157	NA	NA	0.00	0.00	NA	NA	NA	NA	NA	NA
Mandal (2014)	Caucasian	Prostate	YES	Hospital	Retrosp.	84	78	59.8	57.2	1.00	1.00	NA	NA	NA	NA	NA	NA
Mandal (2014)	African-American	Prostate	YES	Hospital	Retrosp.	80	62	67.9	64.0	1.00	1.00	NA	NA	NA	NA	NA	NA
Cil (2014)	Mixed	Thyroid	YES	Hospital	Retrosp.	190	216	47.2	46.0	0.23	0.26	25.55	25.70	0.34	0.32	NA	NA
Tindall (2012)	Caucasian	Prostate	YES	Population	Prosp.	818	734	NA	NA	1.00	1.00	NA	NA	NA	NA	NA	NA
Giannitrapani (2011)	Caucasian	Liver	YES	Population	Retrosp.	95	98	NA	NA	0.53	NA	NA	NA	NA	NA	NA	NA
Giannitrapani (2011)	Caucasian	Liver	YES	Population	Retrosp.	105	98	NA	NA	0.63	NA	NA	NA	NA	NA	NA	NA
Gaur (2011)	Mixed	Oral	YES	Hospital	Prosp.	140	120	51.4	51.4	0.85	0.85	NA	NA	NA	NA	NA	NA
Abuli (2011)	Caucasian	Colorectal	YES	Population	Prosp.	1405	1388	NA	NA	NA	NA	NA	NA	NA	NA	NA	NA
Cacev (2010)	Caucasian	Colorectal	NA	Population	Retrosp.	160	160	64.5	63.1	0.53	0.54	NA	NA	NA	NA	NA	NA
Ognjanovic (2010)	Mixed	Colorectal	YES	Population	Retrosp.	271	539	62.5	62.0	0.68	0.69	26.90	26.70	0.14	0.10	0.45	0.41
Hawken (2010)	Mixed	Colorectal	NA	Population	Retrosp.	1133	1125	NA	NA	NA	NA	NA	NA	NA	NA	NA	NA
Dossus (2010)	Mixed	Breast	YES	Population	Prosp.	6292	8135	63.1	63.1	0.00	0.00	NA	NA	NA	NA	NA	NA
Dossus (2010)	Mixed	Prostate	YES	Population	Prosp.	8008	8604	68.4	68.4	1.00	1.00	NA	NA	NA	NA	NA	NA
Tsilidis (2009)	Mixed	Colorectal	YES	Population	Prosp.	208	318	62.8	62.8	0.46	0.45	26.30	26.00	0.13	0.13	NA	NA
Ozgen (2009)	Mixed	Thyroid	NA	Hospital	Retrosp.	42	340	43.1	43.8	0.19	0.19	NA	NA	NA	NA	NA	NA
Ognjanovic (2009)	Mixed	Liver	YES	Population	Retrosp.	120	230	60.5	59.5	0.68	0.60	NA	NA	0.42	0.28	0.71	0.77
Gangwar (2009)	Asian	Cervical	YES	Hospital	Retrosp.	160	200	45.0	46.0	0.00	0.00	NA	NA	0.34	0.10	0.06	0.01
Falleti (2009)	Caucasian	Liver	NO	Population	Retrosp.	219	236	53.0	46.0	0.70	0.70	NA	NA	NA	NA	NA	NA
Cherel (2009)	Caucasian	Breast	NA	Hospital	Prosp.	293	112	NA	NA	0.00	0.00	NA	NA	NA	NA	NA	NA
Vasku (2009)	Caucasian	Colorectal	YES	Hospital	Retrosp.	102	101	68.0	68.1	0.77	0.58	NA	NA	NA	NA	NA	NA
Talar-Wojnarowska (2009)	Caucasian	Pancreatic	YES	Hospital	Retrosp.	97	50	NA	NA	0.57	0.57	NA	NA	NA	NA	NA	NA
Slattery (2009)	Mixed	Colorectal	NA	Population	Retrosp.	1839	2014			0.55	0.54	NA	NA	NA	NA	NA	NA
Andrie (2009)	Caucasian	Lymphoma	YES	Hospital	Retrosp.	81	81	NA	NA	NA	NA	NA	NA	NA	NA	NA	NA
Aladzsity (2009)	Caucasian	Myeloma	YES	Hospital	Retrosp.	97	99	65.0	68.0	0.35	0.45	NA	NA	NA	NA	NA	NA
Birmann (2009)	Mixed	Myeloma	NA	Population	Prosp.	82	159	NA	NA	NA	NA	NA	NA	NA	NA	NA	NA
Wilkening (2008)	Caucasian	Colorectal	YES	Population	Prosp.	308	585	56.8	56.8	0.44	0.44	NA	NA	NA	NA	NA	NA
Vairaktaris (2008)	Caucasian	Oral	YES	Population	Retrosp.	162	168	58.5	54.7	0.80	0.75	NA	NA	NA	NA	NA	NA
Upadhyay (2008)	Asian	Esophageal	YES	Hospital	Retrosp.	168	201	56.8	53.7	0.74	0.75	NA	NA	0.82	NA	0.37	NA
Slattery (2008) b	Caucasian	Breast	YES	Population	Retrosp.	1176	1330	NA	NA	0.00	0.00	NA	NA	NA	NA	NA	NA
Slattery (2008) a	Mixed	Breast	YES	Population	Retrosp.	576	727	NA	NA	0.00	0.00	NA	NA	NA	NA	NA	NA
Kesarwani (2008)	Asian	Prostate	YES	Population	Retrosp.	200	200	62.5	59.5	1.00	1.00	NA	NA	0.32	0.30	0.60	0.69
Crusius (2008)	Caucasian	Gastric	YES	Population	Prosp.	439	1138	NA	NA	NA	NA	NA	NA	NA	NA	NA	NA
Colakogullari (2008)	Mixed	Lung	YES	Population	Prosp.	44	58	60.0	NA	0.91	0.51	NA	NA	NA	NA	NA	NA
Bao (2008)	Asian	Prostate	YES	Hospital	Retrosp.	136	120	62.8	62.3	1.00	1.00	NA	NA	NA	NA	NA	NA
Vogel (2008)	Caucasian	Lung	YES	Population	Retrosp.	403	744	NA	NA	0.54	0.56	NA	NA	0.73	0.35	NA	NA
Kury (2008)	Caucasian	Colorectal	YES	Hospital	Retrosp.	1023	1121	65.7	61.9	0.62	0.54	NA	NA	NA	NA	NA	NA
Ennas (2008)	Caucasian	Leukaemia	NO	Population	Retrosp.	40	113	61.8	56.5	0.73	0.40	NA	NA	NA	NA	NA	NA
Ahirwar (2008)	Asian	Bladder	YES	Population	Prosp.	136	200	61.6	58.3	0.88	0.88	NA	NA	NA	NA	NA	NA
Vishnoi (2007)	Asian	Gallbladder	YES	Population	Retrosp.	45	82	49.4	50.0	1.00	1.00	NA	NA	NA	NA	NA	NA
Vishnoi (2007)	Asian	Gallbladder	YES	Population	Retrosp.	79	118	49.4	50.0	0.00	0.00	NA	NA	NA	NA	NA	NA
Litovkin (2007)	Caucasian	Breast	YES	Population	Prosp.	73	143	55.0	33.0	0.00	0.49	NA	NA	NA	NA	NA	NA
Litovkin (2007)	Caucasian	Leiomyoma	YES	Population	Prosp.	60	143	37.0	33.0	0.00	0.49	NA	NA	NA	NA	NA	NA
Gonullu (2007)	Mixed	Breast	YES	Population	Retrosp.	38	24	47.0	39.0	0.00	0.00	NA	NA	NA	NA	NA	NA
Vogel (2007)	Caucasian	Breast	YES	Population	Prosp.	361	361	NA	NA	0.00	0.00	25.00	25.00	0.34	0.36	NA	NA
Vogel (2007)	Caucasian	Colorectal	YES	Population	Prosp.	355	753	59.0	56.0	0.56	0.56	26.00	26.00	0.37	0.35	NA	NA
Slattery (2007)	Mixed	Colorectal	YES	Population	Retrosp.	1583	1979	NA	NA	NA	NA	NA	NA	NA	NA	NA	NA
Slattery (2007)	Mixed	Colorectal	YES	Population	Retrosp.	797	1011	NA	NA	NA	NA	NA	NA	NA	NA	NA	NA
Nearman (2007)	Mixed	Leukaemia	NA	Hospital	Retrosp.	28	362	NA	NA	NA	NA	NA	NA	NA	NA	NA	NA
Gatti (2007)	Mixed	Gastric	NA	Hospital	Retrosp.	56	56	NA	NA	NA	NA	NA	NA	NA	NA	NA	NA
Duch (2007)	Mixed	Myeloma	YES	Hospital	Retrosp.	52	60	58.5	59.3	0.42	0.60	NA	NA	NA	NA	NA	NA
Deans (2007)	Caucasian	Gastric	NA	Population	Retrosp.	203	224	71.0	39.2	0.66	0.53	NA	NA	NA	NA	NA	NA
Berkovic (2007)	Caucasian	Gastric	YES	Hospital	Retrosp.	80	162	80.0	46.5	0.48	0.48	NA	NA	NA	NA	NA	NA
Vairaktaris (2006)	Caucasian	Oral	YES	Population	Retrosp.	162	156	58.5	55.5	0.80	0.77	NA	NA	NA	NA	NA	NA
Theodoropoulos (2006)	Caucasian	Colorectal	YES	Population	Prosp.	222	200	64.7	62.7	0.58	0.60	NA	NA	NA	NA	NA	NA
Nogueira (2006)	Mixed	Cervical	YES	Population	Retrosp.	56	253	52.2	54.0	0.00	0.00	NA	NA	NA	NA	NA	NA
Michaud (2006)	Mixed	Prostate	YES	Population	Prosp.	503	652	67.1	66.6	1.00	1.00	NA	NA	0.08	0.11	NA	NA
Kamangar (2006)	Caucasian	Gastric	YES	Population	Prosp.	110	203	58.5	59.0	1.00	1.00	NA	NA	1.00	1.00	NA	NA
Gonzalez-Zuloeta (2006)	Caucasian	Breast	NA	Population	Prosp.	171	3651	67.8	70.8	0.00	0.00	26.70	27.10	NA	NA	NA	NA
Balasubramanian (2006)	Caucasian	Breast	NA	Hospital	Prosp.	197	490	63.0	57.0	0.00	0.00	NA	NA	NA	NA	NA	NA
Rothman (2006)	Caucasian	Lymphoma	NA	Population	Retrosp.	2658	3068	NA	NA	NA	NA	NA	NA	NA	NA	NA	NA
Lan (2006)	Mixed	Lymphoma	YES	Population	Retrosp.	518	597	NA	NA	0.00	0.00	NA	NA	NA	NA	NA	NA
Gunter (2006)	Mixed	Colorectal	YES	Hospital	Retrosp.	244	231	60.0	57.0	0.78	0.64	26.50	25.80	0.11	0.05	NA	NA
Gaustadnes (2006)	Caucasian	Colorectal	YES	Population	Retrosp.	230	540	NA	NA	NA	NA	NA	NA	NA	NA	NA	NA
Cozen (2006)	Mixed	Myeloma	YES	Population	Retrosp.	150	112	61.0	NA	0.61	0.58	NA	NA	NA	NA	NA	NA
Seifart (2005)	Caucasian	Lung	NA	Population	Retrosp.	182	243	63.3	37.9	0.88	0.55	NA	NA	0.98	0.40	NA	NA
Migita (2005)	Asian	Liver	NO	Hospital	Prosp.	48	188	62.5	51.5	0.81	0.68	NA	NA	NA	NA	NA	NA
Leibovici (2005)	Caucasian	Bladder	YES	Hospital	Prosp.	465	450	NA	NA	NA	NA	NA	NA	0.74	0.53	NA	NA
Hefler (2005)	Caucasian	Breast	YES	Hospital	Retrosp.	269	227	54.9	53.3	0.00	0.00	NA	NA	NA	NA	NA	NA
Basturk (2005)	Mixed	Renal cell	YES	Population	Retrosp.	29	50	NA	NA	0.05	0.56	NA	NA	NA	NA	NA	NA
Snoussi (2005)	Caucasian	Breast	NA	Population	Prosp.	305	200	50.0	46.0	0.01	0.05	NA	NA	NA	NA	NA	NA
Skerrett (2005)	Mixed	Breast	NA	Population	Retrosp.	88	102	49.2	NA	0.00	0.00	NA	NA	NA	NA	NA	NA
Mazur (2005)	Caucasian	Myeloma	NA	Hospital	Retrosp.	54	50	62.0	NA	0.43	0.58	NA	NA	NA	NA	NA	NA
Festa (2005)	Caucasian	Basal cell	NA	Population	Prosp.	241	260	NA	NA	NA	NA	NA	NA	NA	NA	NA	NA
Cordano (2005)	Caucasian	Lymphoma	YES	Population	Retrosp.	408	349	NA	NA	NA	NA	NA	NA	NA	NA	NA	NA
Campa (2005)	Caucasian	Lung	NA	Hospital	Retrosp.	1995	1982	NA	NA	NA	NA	NA	NA	NA	NA	NA	NA
Zhang (2004)	Caucasian	Basal cell	YES	Hospital	Retrosp.	241	260	50.0	48.0	0.58	0.54	NA	NA	NA	NA	NA	NA
Smith (2004)	Caucasian	Breast	NO	Population	Retrosp.	144	263	59.6	40.3	0.00	0.00	NA	NA	NA	NA	NA	NA
Campa (2004)	Caucasian	Lung	YES	Population	Prosp.	250	214	63.1	64.8	0.71	0.75	NA	NA	0.73	0.91	NA	NA
Bushley (2004)	Mixed	Ovarian	YES	Population	Retrosp.	182	219	NA	NA	0.00	0.00	NA	NA	NA	NA	NA	NA
Landi (2003)	Mixed	Colorectal	YES	Hospital	Retrosp.	377	326	NA	NA	0.60	0.53	NA	NA	0.15	0.18	0.67	0.56
El-Omar (2003)	Mixed	Esophageal	YES	Population	Retrosp.	161	210	65.5	66.0	0.87	0.85	NA	NA	0.40	0.24	0.87	0.85
El-Omar (2003)	Mixed	Gastric	YES	Population	Retrosp.	314	210	68.0	66.0	0.81	0.85	NA	NA	0.30	0.24	0.75	0.85
Hwang (2003)	Asian	Gastric	NA	Hospital	Retrosp.	30	30	NA	NA	NA	NA	NA	NA	NA	NA	NA	NA
Hwang (2003)	Caucasian	Gastric	NA	Hospital	Retrosp.	30	30	NA	NA	NA	NA	NA	NA	NA	NA	NA	NA
Howell (2003)	Caucasian	Melanoma	NA	Hospital	Prosp.	153	208	NA	NA	NA	NA	NA	NA	NA	NA	NA	NA
Zheng (2000)	Caucasian	Myeloma	NA	Population	Retrosp.	73	129	67.0	NA	0.45	NA	NA	NA	NA	NA	NA	NA

Abbreviations: BMI, body mass index; cont’s, controls; NA, not available; Prosp., prospective design; Retrosp., retrospective design.

**Table 2:** Mean circulating IL-6 across IL-6 gene -174G/C genotypes in this meta-analysis.

**Author (year)**	**Ethnicity**	**Status**	**Sample size**	**Circulating IL-6 (pg/mL) across -174G/C genotypes**					
				**Mean (GG)**	**SD (GG)**	**Mean (GC)**	**SD (GC)**	**Mean (CC)**	**SD (CC)**
Malaponte (2013)	Caucasian	Cancer patients	130	22.10	4.30	11.90	2.60	5.30	1.50
Malaponte (2013)	Caucasian	Cancer patients	190	7.20	2.20	12.00	1.70	4.50	1.10
Malaponte (2013)	Caucasian	Healthy controls	215	2.80	0.70	2.70	0.50	2.60	0.70
Giannitrapani (2011)	Caucasian	Cancer patients	67	2.20	2.86	2.30	1.70	2.90	0.60
Giannitrapani (2011)	Caucasian	Cancer patients	80	4.80	4.25	3.60	3.00	0.87	1.97
Ognjanovic (2010)	Mixed	Cancer patients and controls	806	1.90	0.05	2.23	2.69	2.35	1.91
Talar-Wojnarowska (2009)	Caucasian	Cancer patients	97	65.00	10.00	27.00	10.00	33.00	10.00
Berkovic (2007)	Caucasian	Cancer patients	80	3.07	1.03	4.31	2.98	7.51	4.33
Belluco (2003)	Caucasian	Cancer patients	62	0.32	0.62	0.14	0.13	0.14	0.13

Abbreviations: SD, standard deviation.

**Table 3:** Overall and stratified risk estimates of IL-6 gene -174G/C variant for cancer risk under three genetic models.

**Groups**	**Studies**	**Allelic model**		**Genotypic model**		**Dominant model**	
		**OR; 95% CI; P**	***I***^**2**^	**OR; 95% CI; P**	***I***^**2**^	**OR; 95% CI; P**	***I***^**2**^
**Overall**	87	1.02; 0.98–1.07; 0.290	66.6%	1.07; 0.99–1.16; 0.103	54.0%	1.02; 0.97–1.08; 0.465	65.1%
**Ethnicity**							
** Caucasian**	46	1.05; 0.98–1.12; 0.138	72.2%	1.09; 0.97–1.23; 0.136	59.5%	1.07; 0.98–1.17; 0.155	71.4%
** Asian**	9	1.03; 0.75–1.42; 0.870	68.8%	1.95; 1.19–3.20; 0.009	17.9%	0.90; 0.66–1.22; 0.483	52.7%
** Mixed**	31	0.98; 0.93–1.03; 0.374	50.6%	0.97; 0.88–1.06; 0.450	27.5%	0.97; 0.91–1.04; 0.430	53.0%
**Sample size**							
** <500**	54	1.03; 0.92–1.15; 0.615	73.7%	1.17; 0.94–1.45; 0.163	61.8%	1.03; 0.89–1.20; 0.654	72.7%
** >=500**	33	1.01; 0.98–1.04; 0.730	43.5%	1.02; 0.96–1.08; 0.615	29.2%	1.01; 0.96–1.05; 0.817	41.4%
**Cancer type**							
** Myeloma**	6	1.06; 0.89–1.28; 0.496	0.0%	1.13; 0.72–2.50; 0.592	0.0%	1.09; 0.84–1.40; 0.521	0.0%
** Gastric**	8	1.01; 0.79–1.28; 0.960	68.5%	1.12; 0.81–1.54; 0.498	28.2%	1.04; 0.72–1.51; 0.819	74.1%
** Colorectal**	16	1.00; 0.93–1.07; 0.941	63.3%	0.99; 0.88–1.13; 0.914	51.7%	1.01; 0.92–1.11; 0.850	58.1%
** Lung**	5	1.03; 0.95–1.11; 0.530	3.6%	1.06; 0.91–1.22; 0.463	0.0%	1.03; 0.86–1.25; 0.743	41.8%
** Breast**	14	0.99; 0.93–1.05; 0.716	44.0%	1.02; 0.89–1.16; 0.824	11.3%	0.99; 0.89–1.09; 0.773	58.2%
** Lymphoma**	4	1.00; 0.95–1.07; 0.888	0.0%	1.01; 0.89–1.15; 0.855	0.0%	1.00; 0.92–1.10; 0.940	0.0%
** Liver**	5	0.74; 0.61–0.89; 0.001	0.0%	0.59; 0.36–0.95; 0.029	0.0%	0.67; 0.52–0.88; 0.004	21.5%
** Prostate**	7	0.95; 0.80–1.14; 0.597	79.4%	0.94; 0.66–1.34; 0.724	76.3%	0.96; 0.81–1.13; 0.609	58.8%
** Oral**	3	1.49; 0.58–3.81; 0.409	95.0%	2.38; 0.34–16.93; 0.385	91.6%	1.98; 0.54–7.26; 0.303	95.4%
**Matched**							
** NA**	23	1.00; 0.94–1.06; 0.961	43.1%	0.99; 0.89–1.10; 0.821	18.1%	1.00; 0.92–1.08; 0.926	41.1%
** YES**	60	1.03; 0.98–1.09; 0.235	71.5%	1.11; 1.00–1.23; 0.044	59.9%	1.03; 0.96–1.11; 0.389	70.8%
** NO**	4	0.99; 0.68–1.45; 0.976	70.4%	1.00; 0.40–2.50; 0.999	75.0%	0.97; 0.69–1.36; 0.846	35.1%
**Control source**							
** Population**	57	1.01; 0.97–1.06; 0.592	61.5%	1.02; 0.94–1.11; 0.622	47.9%	1.02; 0.96–1.08; 0.569	63.4%
** Hospital**	30	1.02; 0.91–1.14; 0.701	73.3%	1.17; 0.96–1.43;.0118	58.9%	1.00; 0.86–1.16; 0.973	68.7%
**Study design**							
** Retrospective**	59	1.02; 0.96–1.08; 0.546	69.0%	1.09; 0.97–1.23; 0.163	54.1%	1.01; 0.93–1.10; 0.751	68.8%
** Prospective**	28	1.03; 0.97–1.09; 0.316	61.6%	1.06; 0.95–1.19; 0.328	55.7%	1.04; 0.96–1.11; 0.350	54.8%

Abbreviations: OR, odds ratio; 95% CI, 95% confidence interval; NA, not available.
