# Supplementary material for: Circulating interleukin-6 and cancer: A meta-analysis using Mendelian randomization
Source: Sci Rep. 2015 Jun 22;5:11394. doi: 10.1038/srep11394 (PMC4476043; doi:10.1038/srep11394)
Supplement: Supplementary Information [file srep11394-s1.pdf]

Circulating interleukin-6 and cancer: A meta-analysis using Mendelian randomization

Geng Tian[1,*,#], Jia Mi[1,*], Xiaodan Wei[1,*], Dongmei Zhao[2], Lingyan Qiao[3], Chunhua Yang[1], Xianglin Li[1], Shuping Zhang[4], Xuri Li[1], Bin Wang[5,#]

Author affiliations:

[1]Medicine and Pharmacy Research Center, Binzhou Medical University, Laishan District, Yantai, Shandong, China; [2]Institute of Anatomy, Binzhou Medical University, Laishan District, Yantai, Shandong, China; [3]Clinic Institute, Binzhou Medical University, Laishan District, Yantai, Shandong, China; [4]Institute of Pharmacology, Binzhou Medical University, Laishan District, Yantai, Shandong, China. [5]Institute of Molecular Imaging, Binzhou Medical University, Laishan District, Yantai, Shandong, China.

Correspondence should be addressed to:

Geng Tian, M.D. Ph.D. or Bin Wang, M.D. Ph.D.

No. 346, Guanhai Road, Laishan District Yantai 264003, Shandong, China.

Tel: 86 0535 6913395; Fax: 86 0535 6913075;

E-mail: tiangengshandong@yeah.net (G.T.) and wangbindoctor2015@163.com (B.W.).

Supplementary PRISMA Checklist

Section/topic	#	Checklist item	Reported section
**TITLE**			
Title	1	Identify the report as a systematic review, meta-analysis, or both.	Title page
**ABSTRACT**			
Structured summary	2	Provide a structured summary including, as applicable: background; objectives; data sources; study eligibility criteria, participants, and interventions; study appraisal and synthesis methods; results; limitations; conclusions and implications of key findings; systematic review registration number.	Abstract
**INTRODUCTION**			
Rationale	3	Describe the rationale for the review in the context of what is already known.	Introduction (1st and 2nd paragraphs)
Objectives	4	Provide an explicit statement of questions being addressed with reference to participants, interventions, comparisons, outcomes, and study design (PICOS).	Introduction (3rd paragraph)
**METHODS**			
Protocol and registration	5	Indicate if a review protocol exists, if and where it can be accessed (e.g., Web address), and, if available, provide registration information including registration number.	Methods/Search strategy
Eligibility criteria	6	Specify study characteristics (e.g., PICOS, length of follow-up) and report characteristics (e.g., years considered, language, publication status) used as criteria for eligibility, giving rationale.	Methods/Inclusion/exclusion criteria
Information sources	7	Describe all information sources (e.g., databases with dates of coverage, contact with study authors to identify additional studies) in the search and date last searched.	Methods/Search strategy
Search	8	Present full electronic search strategy for at least one database, including any limits used, such that it could be repeated.	Methods/Search strategy
Study selection	9	State the process for selecting studies (i.e., screening, eligibility, included in systematic review, and, if applicable, included in the meta-analysis).	Figure 1
Data collection process	10	Describe method of data extraction from reports (e.g., piloted forms, independently, in duplicate) and any processes for obtaining and confirming data from investigators.	Methods/Data extraction
Data items	11	List and define all variables for which data were sought (e.g., PICOS, funding sources) and any assumptions and simplifications made.	Methods/Data extraction and Methods/Quality assessment, Table 1, Table 2 and Supplementary Table S1
Risk of bias in individual studies	12	Describe methods used for assessing risk of bias of individual studies (including specification of whether this was done at the study or outcome level), and how this information is to be used in any data synthesis.	Methods/Statistics
Summary measures	13	State the principal summary measures (e.g., risk ratio, difference in means).	Methods/Statistics
Synthesis of results	14	Describe the methods of handling data and combining results of studies, if done, including measures of consistency (e.g., I^2) for each meta-analysis.	Methods/Statistics
Risk of bias across studies	15	Specify any assessment of risk of bias that may affect the cumulative evidence (e.g., publication bias, selective reporting within studies).	Methods/Statistics
Additional analyses	16	Describe methods of additional analyses (e.g., sensitivity or subgroup analyses, meta-regression), if done, indicating which were pre-specified.	Methods/Statistics

**RESULTS**			
Study selection	17	Give numbers of studies screened, assessed for eligibility, and included in the review, with reasons for exclusions at each stage, ideally with a flow diagram.	Figure 1 and Results/Eligible articles
Study characteristics	18	For each study, present characteristics for which data were extracted (e.g., study size, PICOS, follow-up period) and provide the citations.	Results/Study characteristics, Table 1 and Table 2
Risk of bias within studies	19	Present data on risk of bias of each study and, if available, any outcome level assessment (see item 12).	Table 3 and Results/Prediction of -174G/C variant for cancer risk
Results of individual studies	20	For all outcomes considered (benefits or harms), present, for each study: (a) simple summary data for each intervention group (b) effect estimates and confidence intervals, ideally with a forest plot.	Table 3 and Figure 3
Synthesis of results	21	Present results of each meta-analysis done, including confidence intervals and measures of consistency.	Results/Prediction of -174G/C variant for cancer risk and Results/Changes of circulating IL-6 across -174G/C genotypes
Risk of bias across studies	22	Present results of any assessment of risk of bias across studies (see Item 15).	Table 3 and Figure 2
Additional analysis	23	Give results of additional analyses, if done (e.g., sensitivity or subgroup analyses, meta-regression [see Item 16]).	Results/Predicted causality of circulating IL-6 for cancer and Results/Sensitivity analysis
**DISCUSSION**			
Summary of evidence	24	Summarize the main findings including the strength of evidence for each main outcome; consider their relevance to key groups (e.g., healthcare providers, users, and policy makers).	Discussion (1st paragraph)
Limitations	25	Discuss limitations at study and outcome level (e.g., risk of bias), and at review-level (e.g., incomplete retrieval of identified research, reporting bias).	Discussion (5th paragraph)
Conclusions	26	Provide a general interpretation of the results in the context of other evidence, and implications for future research.	Discussion (6th paragraph)
**FUNDING**			
Funding	27	Describe sources of funding for the systematic review and other support (e.g., supply of data); role of funders for the systematic review.	Grant support

From: Moher D, Liberati A, Tetzlaff J, Altman DG, The PRISMA Group (2009). Preferred Reporting Items for Systematic Reviews and Meta-Analyses: The PRISMA Statement. PLoS Med 6(6): e1000097. doi:10.1371/journal.pmed1000097.

Supplementary Table S1. Quality assessment of all study groups and genotype distributions of *IL-6* gene -174G/C variant

Author (year)	Q1	Q2	Q3	Q4	Q5	Q6	Q7	Quality score	***IL-6* gene -174G/C**						H-W P
									**Cases**			**Controls**			
									**GG**	**GC**	**CC**	**GG**	**GC**	**CC**	
Slattery (2014)	1	2	1	1	1	1	2	9	2011	1556	0	2330	1827	0	0.000
Mandal (2014) a	1	0	0	1	0	1	1	4	50	28	6	26	30	22	0.043
Mandal (2014) b	1	0	0	1	0	2	1	5	58	16	6	48	14	0	0.316
Cil (2014)	2	2	1	1	1	2	2	11	110	63	17	113	85	18	0.722
Tindall (2012)	2	2	1	1	0	2	2	10	275	399	144	238	360	136	1.000
Giannitrapani (2011) a	2	1	1	1	0	2	1	8	66	21	8	51	37	10	0.402
Giannitrapani (2011) b	2	1	1	1	0	2	1	8	63	36	6	51	37	10	0.402
Gaur (2011)	1	2	1	0	0	2	2	8	98	35	7	65	41	14	0.069
Abuli (2011)	1	1	1	0	0	2	2	7	586	635	184	593	623	172	0.672
Cacev (2010)	2	1	1	1	1	2	1	9	64	70	26	68	75	17	0.582
Ognjanovic (2010)	2	1	1	1	1	1	2	9	173	74	22	357	136	43	0.000
Hawken (2010)	2	1	1	1	0	2	2	9	381	557	195	373	539	213	0.461
Dossus (2010) a	1	1	1	1	1	1	2	8	2847	2523	820	3707	3324	1035	0.000
Dossus (2010) b	1	1	1	1	1	1	2	8	3594	3218	1125	3832	3402	1274	0.000
Tsilidis (2009)	1	1	1	1	0	2	2	8	68	93	39	113	170	71	0.627
Ozgen (2009)	1	1	1	1	0	1	2	7	21	14	7	143	171	26	0.009
Ognjanovic (2009)	1	1	1	1	1	1	2	8	71	46	0	103	118	0	0.000
Gangwar (2009)	1	1	1	1	1	2	2	9	107	36	17	142	51	7	0.372
Falleti (2009)	1	2	1	1	0	2	2	9	102	98	19	102	103	31	0.536
Cherel (2009)	2	1	1	1	0	2	2	9	102	131	60	29	58	25	0.695
Vasku (2009)	1	1	1	0	0	2	1	6	32	46	22	31	47	22	0.601
Talar-Wojnarowska (2009)	1	0	1	1	0	2	1	6	33	33	21	22	19	9	0.191
Slattery (2009)	2	1	1	1	0	1	2	8	631	696	246	728	897	347	0.015

Andrie (2009)	1	1	1	1	0	2	2	8	55	23	3	47	30	4	0.777
Aladzsity (2009)	2	2	1	1	0	2	2	10	37	43	17	36	49	14	0.681
Birmann (2009)	2	2	1	1	1	2	2	11	21	46	10	52	82	28	0.655
Wilkening (2008)	2	2	1	1	1	2	2	11	79	163	61	162	297	121	0.481
Vairaktaris (2008)	1	1	1	1	1	2	2	9	42	102	18	90	60	6	0.298
Upadhyay (2008)	1	1	1	1	0	2	2	8	135	28	5	131	64	6	0.586
Slattery (2008) b	2	2	1	1	1	1	2	10	430	741	0	407	917	0	0.000
Slattery (2008) a	2	2	1	1	1	1	2	10	389	459	0	186	265	0	0.000
Kesarwani (2008)	1	1	1	1	0	2	2	8	102	84	14	103	87	10	0.120
Crusius (2008)	2	2	1	1	0	1	2	9	140	224	75	415	517	206	0.044
Colakogullari (2008)	1	1	1	1	1	2	1	8	10	29	5	27	22	9	0.222
Bao (2008)	1	1	1	1	0	0	1	5	136	0	0	120	0	0	NA
Vogel (2008)	2	2	1	1	0	2	2	10	105	202	96	204	361	179	0.437
Kury (2008)	1	1	1	1	0	2	2	8	363	489	171	435	504	182	0.079
Ennas (2008)	2	2	1	1	0	2	2	10	17	16	6	64	43	5	0.506
Ahirwar (2008)	1	1	1	1	0	1	2	7	86	24	26	130	56	14	0.027
Vishnoi (2007) a	1	1	1	1	0	2	2	8	36	9	0	62	18	2	0.618
Vishnoi (2007) b	1	1	1	1	0	2	2	8	61	16	2	91	26	1	0.561
Litovkin (2007) a	1	1	1	1	0	2	2	8	26	39	8	30	39	9	0.490
Litovkin (2007) b	1	1	1	1	0	2	2	8	18	25	17	30	39	9	0.490
Gonullu (2007)	1	1	1	1	0	1	2	7	15	17	6	14	3	7	0.000
Vogel (2007)	1	1	1	1	0	2	2	8	108	167	86	98	177	86	0.728
Vogel (2007)	2	2	1	1	0	2	2	10	98	168	89	204	364	185	0.371
Slattery (2007) a	2	2	1	1	1	1	2	10	631	696	246	728	897	347	0.015
Slattery (2007) b	2	2	1	1	1	2	2	11	321	347	109	411	438	146	0.098
Nearman (2007)	1	1	1	1	0	2	2	8	9	15	4	181	141	40	0.128
Gatti (2007)	1	1	1	1	0	2	2	8	42	13	1	23	27	6	0.642
Duch (2007)	1	1	1	1	0	2	1	7	28	22	2	35	23	2	0.442

Deans (2007)	2	2	1	1	0	2	2	10	71	83	43	79	101	44	0.258
Berkovic (2007)	1	1	1	1	0	2	2	8	25	44	11	69	75	18	0.724
Vairaktaris (2006)	1	1	1	1	0	2	2	8	42	102	18	90	60	6	0.298
Theodoropoulos (2006)	1	1	1	1	0	2	2	8	111	76	35	64	86	50	0.055
Nogueira (2006)	1	1	1	1	1	1	2	8	24	32	0	148	102	3	0.001
Michaud (2006)	2	2	1	1	1	2	2	11	170	223	91	230	293	90	0.832
Kamangar (2006)	2	2	1	1	0	1	2	9	21	54	27	51	58	43	0.004
Gonzalez-Zuloeta (2006)	1	1	1	1	0	2	2	8	55	86	30	1286	1733	632	0.246
Balasubramanian (2006)	1	1	1	1	0	2	2	8	170	244	83	168	235	87	0.759
Rothman (2006)	2	2	1	1	0	2	2	10	1097	1470	499	1277	1658	564	0.506
Lan (2006)	2	2	1	1	0	2	2	10	211	231	68	241	264	85	0.358
Gunter (2006)	1	1	1	1	1	2	2	9	79	90	35	83	81	26	0.385
Gaustadnes (2006)	1	1	1	1	0	2	2	8	64	115	51	184	263	93	0.979
Cozen (2006)	2	2	1	1	0	1	2	9	85	61	0	75	50	0	0.005
Seifart (2005)	1	1	1	1	0	2	2	8	74	82	26	90	107	46	0.163
Migita (2005)	1	1	1	1	0	0	1	5	48	0	0	188	0	0	NA
Leibovici (2005)	1	1	1	1	0	2	2	8	134	200	110	175	200	68	0.387
Hefler (2005)	1	1	1	1	0	2	2	8	78	139	52	91	105	31	0.935
Basturk (2005)	1	1	1	1	1	1	2	8	15	10	0	27	13	9	0.007
Snoussi (2005)	1	1	1	1	0	2	1	7	199	98	8	150	46	4	0.830
Skerrett (2005)	2	2	1	1	0	2	2	10	74	13	1	88	14	0	0.931
Mazur (2005)	1	1	1	1	0	2	1	7	11	31	12	16	28	6	0.239
Festa (2005)	1	1	1	1	0	2	1	7	57	126	58	62	130	68	0.993
Cordano (2005)	1	1	1	1	0	2	2	8	134	197	77	106	184	59	0.167
Campa (2005)	2	2	1	1	0	2	2	10	629	954	412	615	993	374	0.448
Zhang (2004)	1	1	1	1	0	2	1	7	57	126	58	62	130	68	0.993
Smith (2004)	1	1	1	1	0	2	1	7	57	67	20	79	101	44	0.258
Campa (2004)	2	2	1	1	0	2	2	10	64	111	68	55	105	47	0.818

Bushley (2004)	2	2	1	1	1	1	2	10	143	34	5	163	46	9	0.020
Landi (2003)	2	2	1	1	0	2	2	10	133	180	48	145	133	33	0.761
El-Omar (2003) a	2	2	1	1	0	2	2	10	33	39	18	83	98	28	0.913
El-Omar (2003) b	2	2	1	1	0	2	2	10	55	52	16	83	98	28	0.913
Hwang (2003) a	1	1	1	1	1	0	1	6	30	0	0	30	0	0	NA
Hwang (2003) b	1	1	1	1	1	2	1	8	19	9	2	22	8	0	0.393
Howell (2003)	1	1	1	1	0	2	1	7	48	79	34	79	101	44	0.258
Zheng (2000)	1	1	1	1	0	2	1	7	22	36	15	33	69	26	0.357

Abbreviations: H-W P, P value for Hardy–Weinberg equilibrium test.

For “Q1: Representativeness of cases”, 2 denotes “Consecutive/randomly selected from case population with clearly defined random frame”, 1 denotes “Consecutive/randomly selected from case population without clearly defined random frame or with extensive inclusion criteria” and 0 denotes “Method of selection not described”. For “Q2: Representativeness of controls”, 2 denotes “Controls were consecutive/randomly drawn from the same area (ward/community) as cases with the same criteria”, 1 denotes “Controls were consecutive/randomly drawn from a different area than cases” and 0 denotes “Not described”. For “Q3: Ascertainment of cancer cases”, 1 denotes “Clearly described objective criteria for diagnosis of cancer” and 0 denotes “Not described”. For “Q4: Ascertainment of controls”, 2 denotes “Clinical examinations were performed on controls to prove that controls did not have cancer”, 1 denotes “Article merely stated that controls were subjects who did not have cancer; no proof provided” and 0 denotes “Not described”. For “Q5: Ascertainment of genotyping examination”, 1 denotes “Genotyping done under ‘‘blind’’ conditions” and 0 denotes “Unblinded or not mentioned”. For “Q6: Test for Hardy–Weinberg equilibrium”, 2 denotes “Hardy–Weinberg equilibrium in control group”, 1 denotes “Hardy–Weinberg disequilibrium in control group” and 0 denotes “Hardy–Weinberg equilibrium not checked”. For “Q7: Association assessment”, 2 denotes “Assessed association between genotypes and cancer with appropriate statistic and adjusting confounders”, 1 denotes “Assessed association between genotypes and cancer with appropriate statistic without adjusting confounders” and 0 denotes “Inappropriate statistic used”.
